# Changes to benthic community structure may impact organic matter consumption on Pacific Arctic shelves

**DOI:** 10.1093/conphys/coab007

**Published:** 2021-03-31

**Authors:** Brittany R Jones, Amanda L Kelley, Sarah L Mincks

**Affiliations:** College of Fisheries and Ocean Sciences, University of Alaska Fairbanks, AK 99775, USA

**Keywords:** Bivalves, macrofauna, metabolism, oxygen consumption, Pacific Arctic, respiration

## Abstract

Changes in species composition and biomass of Arctic benthic communities are predicted to occur in response to environmental changes associated with oceanic warming and sea-ice loss. Such changes will likely impact ecosystem function, including flows of energy and organic material through the Arctic marine food web. Oxygen consumption rates can be used to quantify differences in metabolic demand among species and estimate the effects of shifting community structure on benthic carbon consumption. Closed-system respirometry using non-invasive oxygen optodes was conducted onboard the *R/V Sikuliaq* in June 2017 and 2018 on six dominant species of benthic macrofauna from the northern Bering and southern Chukchi Sea shelves, including five bivalve species (*Macoma* sp*.*, *Serripes groenlandicus*, *Astarte* sp*.*, *Hiatella arctica* and *Nuculana pernula*) and one amphipod species (*Ampelisca macrocephala*). Results revealed species-specific respiration rates with high metabolic demand for *S. groenlandicus* and *A. macrocephala* compared to that of the other species. For a hypothetical 0.1-g ash-free dry mass individual, the standard metabolic rate of *S. groenlandicus* would be 4.3 times higher than that of *Astarte* sp. Overall, carbon demand ranged from 8 to 475 μg C individual^−1^ day^−1^ for the species and sizes of individuals measured. The allometric scaling of respiration rate with biomass also varied among species. The scaling coefficient was similar for *H. arctica*, *A. macrocephala* and *Astarte* sp., while it was high for *S. groenlandicus* and low for *Macoma* sp. These results suggest that observed shifts in spatial distribution of the dominant macrofaunal taxa across this region will impact carbon demand of the benthic community. Hence, ecosystem models seeking to incorporate benthic system functionality may need to differentiate between communities that exhibit different oxygen demands.

## Introduction

Climate change is impacting Arctic marine ecosystems at a rapid pace. Warming temperatures and declining sea ice (IPCC, 2014; Wang and Overland, 2015; Huntington *et al.*, 2020) are resulting in ecosystem-wide changes in the timing and magnitude of primary production (Arrigo and van Dijken, 2015; Selz *et al.*, 2018), secondary production (Ringuette *et al.*, 2002), the strength of pelagic–benthic coupling (Grebmeier *et al.*, 2006; Moore and Stabeno, 2015) and benthic community structure and function (Grebmeier *et al.*, 2018). These changes are likely to affect metabolic demand of Arctic marine invertebrates and, in turn, the cycling of organic matter in sediments and subsequent exchanges with the water column.

Oxygen consumption rates (*M*O_2_) provide an estimate of metabolic activity in aerobic organisms and serve as a proxy for organic matter consumption and energy flow through the benthic food web. Metabolic rates vary among individuals due to a variety of factors, including developmental stage (Yagi *et al.*, 2010), age (Sukhotin and Pörtner, 2001; Glazier *et al.*, 2015) and body size (Kleiber, 1932). For instance, *M*O_2_ increases with body size in a relatively predictable manner described by the ‘3/4-power law’, wherein the relationship between *M*O_2_ and body size is quantified by a metabolic scaling coefficient of ~0.75 (Kleiber, 1932); however, many exceptions have been reported (reviewed by Glazier, 2005). In addition to physiological differences among individuals and species, *M*O_2_ also varies with environmental factors, such as temperature (Peck *et al.*, 2002; Clarke and Fraser, 2004; Trigos *et al.*, 2015), pH (Liu and He, 2012; Saavedra *et al.*, 2018) and food availability (Brockington and Clarke, 2001; Sejr *et al.*, 2004). Many of these environmental conditions have already changed or are projected to change under future climate scenarios (IPCC, 2014), potentially resulting in alterations to benthic biomass, taxonomic composition and carbon demand.

Estimates of whole sediment-community oxygen consumption rates are available across the Arctic (reviewed in Bourgeois *et al*., 2017; Grebmeier *et al.*, 2006). However, *M*O_2_ rates of individual species have rarely been reported for the region (Vahl, 1978; Opalinski and Weslawksi, 1989; Sejr *et al.*, 2004; Goethel *et al.*, 2017), hampering efforts to predict how changes in species composition may impact benthic carbon processing rates and ecosystem function (Grebmeier, 2012). In the Pacific Arctic region, the Bering and Chukchi Seas overlie a shallow inflow shelf influenced by distinct water masses: cold, nutrient-rich Anadyr-Bering Sea Water and warm, more nutrient-poor Alaska Coastal Water (Danielson *et al.*, 2017). Flows accelerate through the Bering Strait constriction (Danielson *et al.*, 2014), promoting energetic mixing that locally enhances pelagic primary productivity (Walsh *et al.*, 1989). Downstream of this constriction, the current speeds decline, allowing pelagic production and particle flux to settle to the seafloor (Grebmeier *et al.*, 2015b). Such dynamic oceanographic conditions result in a patchy distribution of benthic organisms, with ecologically important hotspots of high benthic biomass up to 32 g C m^−2^ in the Chirikov Basin and southeast Chukchi Sea (Grebmeier *et al.*, 2015b). These hotspots serve as persistent feeding grounds for marine mammals (Fay, 1982) and birds (Lovvorn *et al.*, 2003). Overall, the benthos accounts for a substantial portion of the total food web production in these regions (Walsh *et al*., 1989), dominated by infaunal bivalves and amphipods (Feder *et al.*, 1994; Grebmeier *et al.*, 2015b).

We quantified metabolic rates of dominant macrofaunal benthos from the northern Bering and southern Chukchi Sea shelves by measuring oxygen consumption rates in laboratory incubations. Experiments were conducted using five bivalve species (*Macoma* sp., *Serripes groenlandicus*, *Astarte* sp., *Hiatella arctica* and *Nuculana pernula*) and one amphipod species (*Ampelisca macrocephala*). These species exhibit diverse life-history strategies and functional traits. For instance, *Astarte* sp., *H. arctica* and *S. groenlandicus* are all suspension feeders, while *N. pernula* is a deposit feeder and *Macoma* sp. is a facultative feeder, capable of switching between deposit and suspension feeding. *Ampelisca macrocephala* is primarily a suspension feeder but can supplement its diet by deposit feeding or consuming small crustaceans. *M*O_2_ was measured for multiple individuals of each species, spanning a range of body sizes in order to establish metabolic scaling relationships for each taxon. Overall, we found species-specific respiration rates and differences in metabolic scaling, which have implications for benthic carbon demand particularly considering altered environmental conditions and shifting species assemblages.

## Methods

### Sampling

**Figure 1:**
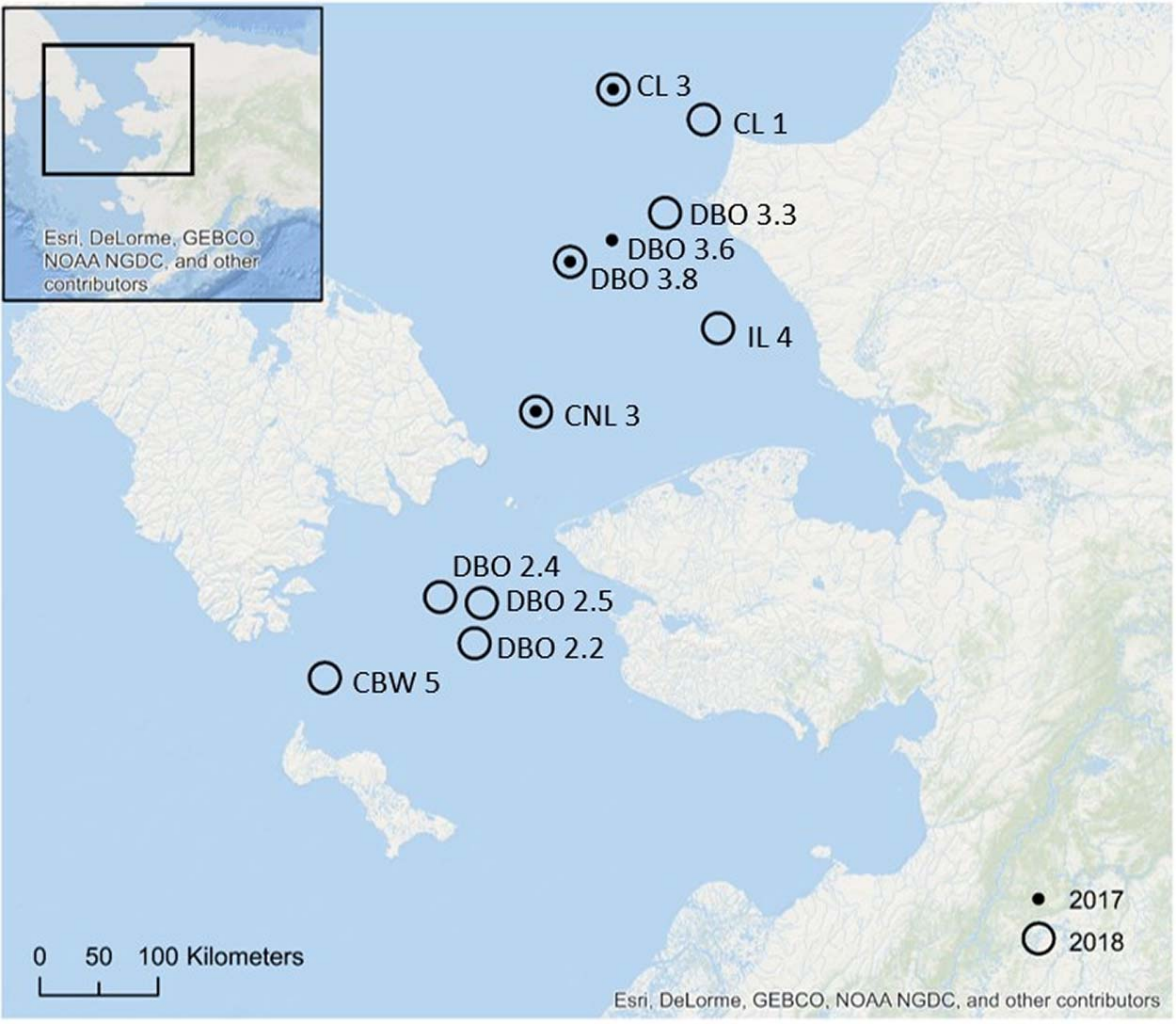
Sampling locations in the northern Bering and southern Chukchi Sea shelves with 2017 in closed circles and 2018 in open circles.

Macrofauna were collected from the northern Bering and southern Chukchi Seas from 13–24 June 2017 and 9–22 June 2018 from the *R/V Sikuliaq* as part of the Arctic Shelf Growth, Advection, Respiration and Deposition (ASGARD) project (Fig. 1, Table 1). Macrofauna were selected from four sampling stations in 2017 and ten stations in 2018 with an average depth of 50 m (ranging from 39 to 59 m). Near-bottom water temperature at sampling location was 2.8 ± 0.7 °C in 2017 and 1.3 ± 0.8 °C in 2018 (Table 1).

**Table 1:** Station locations, depth (m), near-bottom water temperature from CTD (°C), near-bottom water salinity from CTD and numbers of individuals sampled, by taxon

Year	Station	Latitude (°N)	Longitude (°W)	Depth (m)	Temperature (°C)	Salinity	*Macoma sp.*	*Serripes groenlandicus*	*Astarte* sp.	*Hiatella arctica*	*Nuculana pernula*	*Ampelisca macrocephala*
2017							(*N* = 15)	(*N* = 9)				
	CL3	69.03	−168.89	52	2.4	32.8		5				
	CNL3	66.50	−168.96	56	2.2	32.6	9	1				
	DBO3.6	67.90	−168.24	59	3.9	32.9	4	2				
	DBO3.8	67.67	−168.73	50	2.0	32.8	2	1				
2018							(*N* = 11)	(*N* = 17)	(*N* = 6)	(*N* = 5)	(*N* = 12)	(*N* = 14)
	CBW5	64.15	−171.51	46	0.5	32.3			6	2		
	CL1	68.95	−166.91	46	0.0	31.9					3	
	CL3	69.03	−168.89	54	−0.6	32.4		10			6	
	CNL3	66.50	−168.96	56	1.9	32.5	3	5				
	DBO2.2	64.68	−169.10	46	2.4	32.3						6
	DBO2.4	64.96	−169.89	48	1.6	32.4		2				2
	DBO2.5	64.99	−169.14	48	2.8	32.8						6
	DBO3.3	68.19	−167.31	48	0.5	32.5				3		
	DBO3.8	67.67	−168.96	51	1.5	32.8	8					
	IL4	67.40	−165.84	39	2.1	32.5					3	

Individuals were selected from plumb-staff beam trawl, multi-core (MC-800, Ocean Instruments, San Diego) and 0.1-m^2^ Van Veen grab samples. Experiments conducted in 2017 included the bivalves *Macoma* sp. (mostly *M. calcarea*; four small individuals only identified to genus level) and *S. groenlandicus* (Table 1). In 2018, experiments were conducted for additional *Macoma* sp. (one individual only identified to genus) and *S. groenlandicus,* as well as the bivalves *Astarte* sp. (mostly *A. montagui*; one identified only to genus), *N. pernula*, *H. arctica* and the amphipod *A. macrocephala*.

### Respirometry

Closed-system respirometry was performed in a temperature-controlled room onboard the *R/V Sikuliaq*. Non-invasive oxygen optodes (PSt3 oxygen sensor spots; PreSens Precision Sensing GmbH, Germany) were used to measure oxygen concentration inside incubation chambers (Gatti *et al.*, 2002). The sensor spots measure oxygen concentration based on the dynamic fluorescence quenching of a luminophore contained in a polymer matrix and have a detection limit of 0.03% oxygen (15 ppb dissolved oxygen). Factory calibration was used for new sensor spots purchased in 2017 and 2018. For sensor spots that had been used and stored for a year between field seasons, a two-point calibration was performed per manufacturer’s instructions. An aquarium bubbler was used to produce a solution of 100% oxygen air saturation, and a solution of 0% saturation was produced using sodium sulfite and cobalt nitrate (1 g Na_2_SO_3_ and 50 μL of Co(NO_3_)_2_ dissolved in 100 mL of reverse osmosis water to achieve ρ(Co) = 1000 mg L^−1^; in nitric acid 0.5 mol L^−1^).

Prior to the start of each experiment, organisms were rinsed with 0.2-μm filtered seawater and bivalve shells were gently scrubbed with a toothbrush to remove microbial films. Each individual was acclimated to experimental conditions by placing it in an incubation chamber submerged in a water bath, which consisted of a plastic tote filled with 0.2-μm filtered seawater aerated with an aquarium bubbler. The temperature-controlled room was set to a target experimental temperature of 0 °C, but recorded temperatures of the seawater baths averaged 0.6 ± 0.3 °C (standard deviation) in 2017 and 0.9 ± 0.2 °C in 2018. Chambers of various sizes (3.7-, 20-, 60-, 120- and 180-ml glass jars) were used to accommodate different sized individuals such that the estimated body volume of each organism did not exceed ~10% of the chamber volume. After organisms were acclimated to experimental conditions for 12 to 24 hours to minimize stress response, each chamber was sealed ensuring no air bubbles were trapped and re-immersed in a water bath to maintain a constant temperature. Organisms were not fed during the acclimation period or incubations to avoid postprandial effects on metabolic rate (Chapelle *et al.*, 1994). Therefore, these measurements of *M*O_2_ estimate the lower bound of carbon consumption for these organisms, given that metabolic rates typically increase following feeding (i.e. a postprandial effect; Brockington and Clarke, 2001; Sejr *et al.*, 2004). In addition, the species inhabit different sediment depths and exhibit different burrowing behaviours *in situ*, which may have influenced species-specific responses to incubation conditions in the absence of sediment for burrowing.

For each incubation, three control chambers (0.2-μm filtered seawater only) of each chamber size were incubated in the water bath alongside the experimental chambers containing organisms. Oxygen concentration of each chamber was measured every 10 to 60 minutes. Average initial oxygen concentration in all incubations was 344.7 μmol O_2_ L^−1^ (ranging from 310.9 to 371.8 μmol O_2_ L^−1^). The incubation of each individual chamber was terminated when oxygen concentration declined by ~20% of the initial concentration. For some individuals, the target ratio of body volume:chamber volume was exceeded and oxygen levels declined too rapidly to ensure high-quality data; therefore, data were discarded for incubations lasting less than 1.75 hours. Incubations lasted on average 8.2 hours (ranging from 1.8 to 13.1 hours). In 2017, incubations were repeated three times per individual in order to quantify the variability in respiration rate within an individual. Replicate incubations took place on successive days, and between experiments the organisms were held without food in open experimental chambers submerged in the aerated water bath. The respiration chambers were sealed with freshly filtered and aerated seawater for each new incubation. In 2018, triplicate incubations were only performed for a subset of individuals from taxa that were not sampled the previous year.

After incubations, bivalve length was measured at the longest part of the shell (Table 2). Length was not measured for amphipods. Organisms were then individually frozen whole at −80 °C. Samples were transported to the laboratory at the University of Alaska Fairbanks and stored at −20 °C for further analysis. Wet mass was measured on thawed, whole organisms. The volume of water in each chamber was determined based on mass measurements. The thawed organism was placed in its original incubation chamber filled with freshwater, and the mass was determined. The mass of water in each chamber was calculated as the mass of the chamber + organism subtracted from the mass of the chamber + organism + water. This water mass was then converted to water volume using a conversion of 1 mL equals 1 g.

**Table 2:** Power functions of oxygen uptake rate (μmol O_2_ h^−1^) versus ash-free dry mass (g) for taxonomic groups incubated in both years with the R^2^, *P*-values and number of individuals (N) associated with each regression; range of maximum length of individuals incubated in each group (mm); average length of incubated individuals (mm ± standard deviation); maximum achievable length (mm) measured in the field taken from the literature; average length to maximum achievable length ratios; average coefficient of variation (CV ± standard deviation) of oxygen uptake rates (*M*O_2_; μmol O_2_ h^−1^) for individuals incubated in triplicate with number of individuals in parentheses; and average mass-specific *M*O_2_ (μmol O_2_ hr^−1^ g^−1^ ± standard deviation)

Species	Equation	R^2^	*P*-value	N	Length range (mm)	Average length (mm)	Maximum length (mm)*	Avg. length: max. length ratio	Average CV of *M*O_2_	Average mass-specific *M*O_2_ (μmol O_2_ hr^−1^ g^−1^)
*Macoma* sp.	$1.43{mass}^{0.52}$	0.68	<0.001	26	14.60–57.40	29.3 ± 9.1	57	0.51	0.13 ± 0.11 (*n* = 15)	3.1 ± 1.7
2017 *Macoma* sp.	$1.06{mass}^{0.44}$	0.78	<0.001	15	-	-	-	-	-	-
2018 *Macoma* sp.	$1.96{mass}^{0.57}$	0.82	<0.001	11	-	-	-	-	-	-
*S. groenlandicus*	$7.63{mass}^{0.94}$	0.88	<0.001	26	7.40–21.80	14.7 ± 4.6	100	0.15	0.14 ± 0.08 (*n* = 9)	9.5 ± 2.7
*S. groenlandicus* from CNL3	$1.85{mass}^{0.67}$	0.95	0.001	6	-	-	-	-	-	-
*S. groenlandicus* excluding CNL3	$7.75{mass}^{0.89}$	0.97	<0.001	20	-	-	-	-	-	-
*Astarte* sp.	$1.19{mass}^{0.77}$	0.99	<0.001	6	11.05–24.05	19.3 ± 4.2	30	0.64	0.14 ± 0.04 (*n* = 5)	2.1 ± 0.5
*Hiatella arctica*	$2.30{mass}^{0.74}$	0.96	0.003	5	9.04–31.75	19.6 ± 8.0	45	0.43	0.15 ± 0.04 (*n* = 5)	4.2 ± 1.8
*Nuculana pernula*	$1.74{mass}^{0.81}$	0.99	<0.001	12	10.25–31.10	17.6 ± 6.4	30	0.59	0.093 ± 0.04 (*n* = 3)	3.3 ± 0.7
*Ampelisca macrocephala*	$2.87{mass}^{0.77}$	0.93	<0.001	14	-	-	-	-	0.22 ± 0.17 (*n* = 4)	6.6 ± 1.5

^*^(Madsen, 1949; Lubinsky, 1980; Hutchings and Haedrich, 1984; Schaefer et al., 1985; Sejr et al., 2002; Kilada et al., 2007; Sejr and Christensen, 2007)

Dry mass of each individual was determined by drying at 60 °C until constant mass was achieved. Ash-free dry mass (AFDM) was measured by igniting each individual at 500 °C for 6 hrs. For amphipods, dry mass and AFDM were measured on whole individuals. For bivalves, the soft tissue was removed from the shell and dry mass and AFDM were measured for the soft tissue only.

### Data analysis

The linear regression of wet mass versus AFDM was calculated on log-transformed data for all bivalve species taken collectively and for *A. macrocephala* individually, allowing metabolic rates measured here in terms of AFDM to be applied to published estimates of wet biomass from other field studies. Regressions were then expressed as power functions to represent the original data displayed on a log–log scale.

Respiration rates of individuals are typically altered during an initial period of acclimation to the sealed chamber due to handling stress, and the length of this period is variable (Peck and Conway, 2000). The data trend during this acclimation period typically has a different slope than the rest of the incubation. The acclimation period for each individual was thus identified and removed by detecting a breakpoint in the broken-line slope of the linear regression model using a boot-strapped approach, as implemented in the segmented function from the segmented package in R (Muggeo, 2008). Outliers were also identified and removed when standardized residuals were less than −2 or greater than +2. The oxygen consumption rate (*M*O_2_; μmol O_2_ L^−1^ min^−1^) of each individual was then calculated from the linear regression of oxygen concentration versus time. When oxygen concentration significantly changed in the controls, the average rate of the three controls was subtracted from the measured macrofaunal rates of the same incubation to account for background respiration (e.g. by bacteria) or background production. The average rate of change of oxygen concentration in controls was −0.002 μmol O_2_ L^−1^ min^−1^ (ranging from −0.015 to +0.013 μmol O_2_ L^−1^ min^−1^).

Rates were converted to μmol O_2_ hr^−1^ based on the volume of water contained in each incubation chamber. To model the relationship between *M*O_2_ and AFDM, regressions were calculated on log-transformed data for each taxonomic group:

$$ \log{M\mathrm{O}}_2=\mathrm{b}\ast \log \mathrm{M}+\log \mathrm{a}, $$

where *M*O_2_ is the respiration rate (standard metabolic rate), b is the slope, M is the AFDM and log a is the y-intercept. The y-intercept (log a) is the metabolic constant and reflects differences in the magnitude of the respiration rate among species. The slope (b) is the metabolic scaling coefficient, relating respiration rate to biomass. Regression equations were then expressed as a power function to represent the original data plotted on a log–log scale:

$$ {M\mathrm{O}}_2=\mathrm{a}{\mathrm{M}}^{\mathrm{b}}, $$

where a is the y-intercept at x = 1 on the log–log scale, M is the AFDM and b is the slope. To estimate carbon consumption required to support standard metabolic demand, *M*O_2_ was converted to units of carbon respired (μg C individual^−1^ day^−1^) based on a respiratory quotient of 0.8 (Witte and Graf, 1996; Kędra *et al.*, 2010).

Mass-specific metabolic rates were calculated by dividing the oxygen uptake rate of each individual by its respective AFDM. Linear regressions on log-transformed data were also calculated for the relationship between mass-specific oxygen uptake rate and AFDM for each taxonomic group and expressed as a power function.

Differences in the intercepts and the slopes of the linear models among species and between years were examined with analysis of covariance (ANCOVA) and Tukey’s post hoc test. All analyses were performed in R Studio, and the glht function from the multcomp package was used for Tukey’s post hoc (Hothorn *et al.*, 2008). For all comparisons, α = 0.05.

Ratios of average measured body length (our study) to maximum length achievable in the field (from the literature) were also calculated to illustrate the potential relationship between metabolic demand and age.

## Results

Wet mass and AFDM were strongly related for all bivalve species taken collectively and for *A. macrocephala* individually; therefore, oxygen uptake rates are presented relative to AFDM. The mass conversion relationship was y = 0.09x^1.00^ (*n* = 75, R^2^ = 0.95, *P* < 0.001) for bivalves (Supplementary Figure 1a) and y = 0.14x^0.69^ (*n* = 14, R^2^ = 0.90, *P* < 0.001) for *A. macrocephala* (Supplementary Figure 1b), where y is AFDM (g) and x is wet mass (g). Replicate incubations conducted with the same individuals showed little variability in *M*O_2_ based on low coefficients of variation (CV; Table 2), with no consistent increasing or decreasing trend in *M*O_2_ over the three days of incubations.

**Figure 2:**
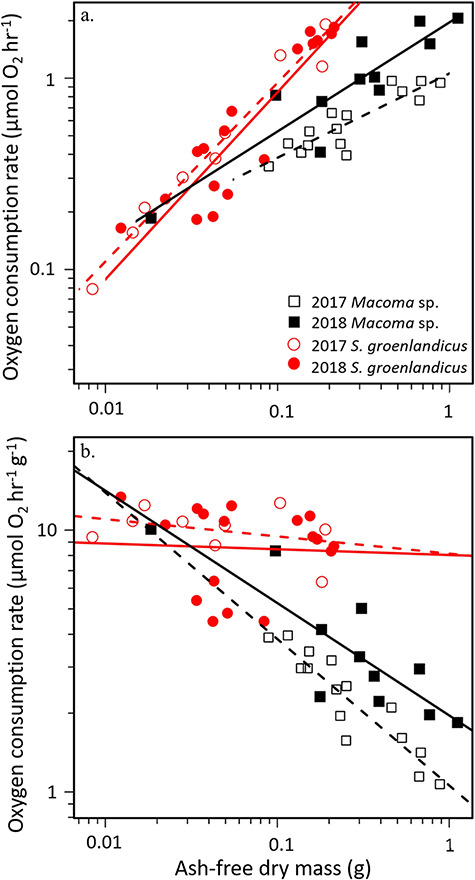
(a) Oxygen uptake rate (μmol O_2_ h^−1^) and (b) mass-specific oxygen uptake rate (μmol O_2_ h^−1^ g^−1^) versus ash-free dry mass (g) for 2017 *Macoma* sp. (black open squares, *n* = 15), 2018 *Macoma* sp. (black closed squares, *n* = 11), 2017 *S. groenlandicus* (red open circles, *n* = 9) and 2018 *S. groenlandicus* (red closed circles, *n* = 17). Dotted lines represent regressions of each species in 2017 and solid lines represent regressions in 2018.

*Macoma* sp. and *S. groenlandicus* were incubated in both years, with slight differences in the average incubation temperature (0.6 ± 0.3 °C in 2017 and 0.9 ± 0.2 °C in 2018). The slopes (F_1,22_ = 0.09, *P* = 0.77) and intercepts (F_1,23_ = 1.01, *P* = 0.32) of the regression relationships relating *M*O_2_ and AFDM were not significantly different between years for *S. groenlandicus* (Fig. 2), indicating no interannual variation, even with the small difference in temperature. Therefore, a single regression is reported for *S. groenlandicus* (Table 2). The slopes (F_1,22_ = 1.33, *P* = 0.26) for *Macoma* sp. were not significantly different between years; however, the intercepts (F_1,23_ = 21.30, *P* < 0.001) were significantly higher in 2018 compared to 2017 and separate regression relationships are reported for each year (Table 2). For *Macoma* sp., we thus present the regression relationships for each year separately, as well as the pooled 2017 and 2018 data which provides an average value for ease of comparison among species.

**Figure 3:**
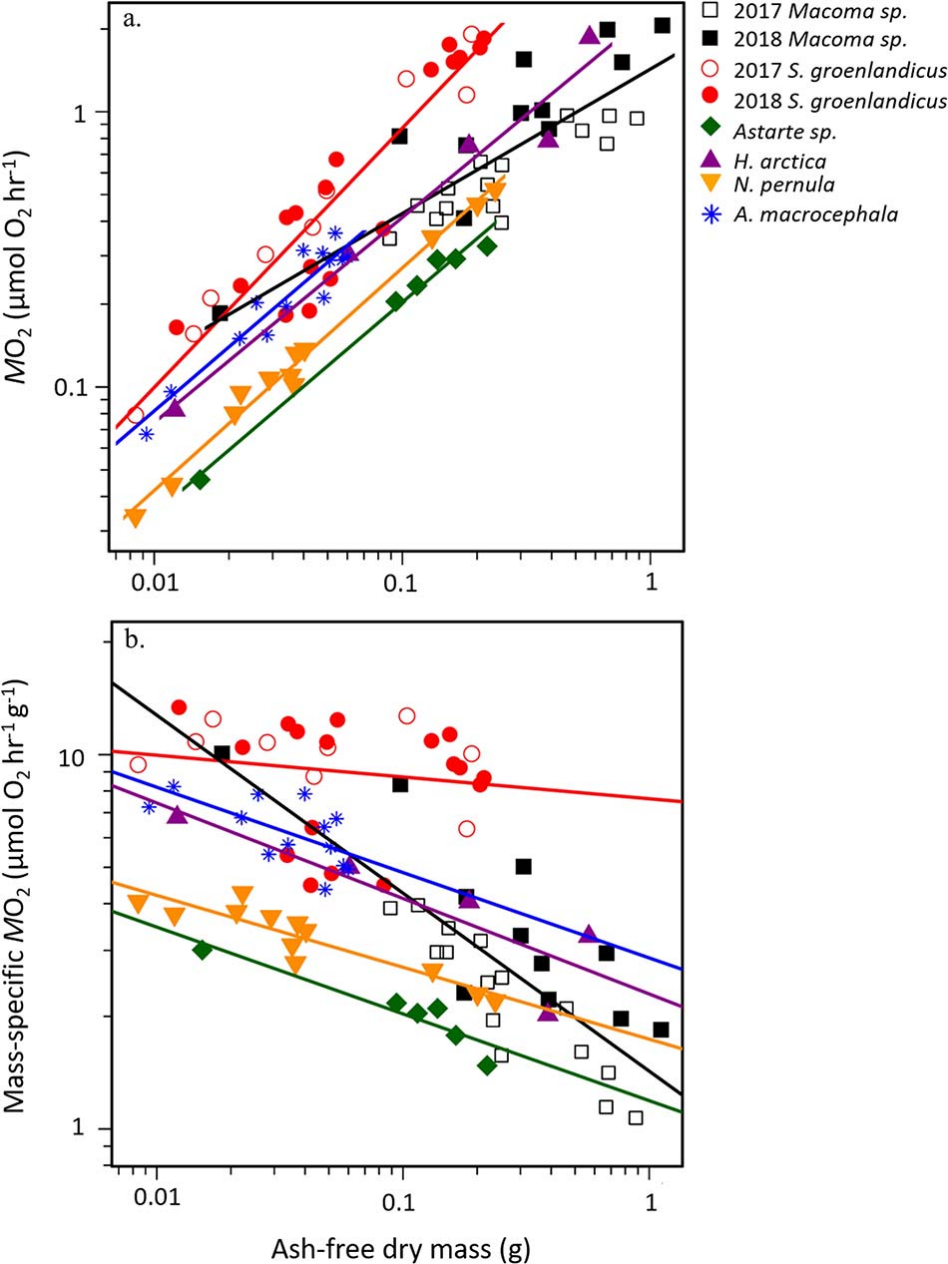
(a) Oxygen uptake rate (μmol O_2_ h^−1^) and (b) mass-specific oxygen uptake rate (μmol O_2_ h^−1^ g^−1^) versus ash-free dry mass (g) for pooled *Macoma* sp. (2017 in open black squares, *n* = 15; 2018 individuals in closed black squares, *n* = 11); pooled *S. groenlandicus* (2017 individuals in open red circles, *n* = 9; 2018 individuals in closed red circles, *n* = 17); and 2018 *Astarte* sp. (green diamonds, *n* = 6), *H. arctica* (purple upright triangles, *n* = 5), *N. pernula* (yellow upside-down triangles, *n* = 12) and *A. macrocephala* (blue asterisks, *n* = 14).

There were significant differences in the slopes (F_5,81_ = 4.47, *P* = 0.001) and intercepts (F_5,86_ = 43.40, *P* < 0.001) for the regressions relating *M*O_2_ and AFDM (Fig. 3a) for all six species (pooled among years). The slope of *S. groenlandicus* was significantly higher than that of *Astarte* sp., *Macoma* sp., *H. arctica* and *N. pernula* (Table 3). Although the slope of *A. macrocephala* (0.77) was lower than that of *N. pernula* (0.81) and the same as that of *Astarte* sp., the standard error of the parameter estimate for *A. macrocephala* was high (0.06), likely reducing the discriminatory power of the post hoc test. The difference was greatest between the slopes of *Macoma* sp. and *S. groenlandicus*, both of which deviated from the ¾-power law for metabolic scaling coefficients (Table 2). The post hoc test showed the intercept of *S. groenlandicus* was significantly higher than that of the other 5 species (Table 4). Over the range of sizes of individuals incubated, *M*O_2_ of *S. groenlandicus* was consistently higher than that of *A. macrocephala*, *H. arctica*, *N. pernula* and *Astarte* sp. (Fig. 3). The intercept for *A. macrocephala* was significantly higher than that of *Macoma* sp., *Astarte* sp. and *N. pernula* (Table 4). Additionally, the intercepts of *H. arctica* and *Macoma* sp. were significantly higher than that of *Astarte* sp. (Table 4).

**Table 3:** Tukey post hoc test statistic (*t*-value) for significant comparisons of slopes of the linear regressions of log-transformed oxygen uptake rate (*M*O_2_; μmol O_2_ h^−1^) versus log-transformed ash-free dry mass (g). See Table 2 for equations and Fig. 3a for plots

Comparison	*t*-value	*P*-value
*S. groenlandicus* > *Macoma* sp.	10.41	<0.001
*S. groenlandicus* > *Astarte* sp.	4.69	<0.001
*S. groenlandicus* > *H. arctica*	3.84	0.003
*S. groenlandicus* > *N. pernula*	4.23	<0.001

**Table 4:** Tukey post hoc test statistic (*t*-value) for significant comparisons of y-intercepts of the linear regressions of log-transformed oxygen uptake rate (μmol O_2_ h^−1^) versus log-transformed ash-free dry mass (g). See Table 2 for equations and Fig. 3a for plots

Comparison	*t*-value	*P*-value
*S. groenlandicus* > *Macoma* sp.	10.35	<0.001
*S. groenlandicus* > *Astarte* sp.	10.20	<0.001
*S. groenlandicus* > *N. pernula*	10.56	<0.001
*S. groenlandicus* > *H. arctica*	4.64	<0.001
*S. groenlandicus* > *A. macrocephala*	4.86	<0.001
*A. macrocephala* > *Macoma* sp.	3.34	0.014
*A. macrocephala* > *Astarte* sp.	5.83	<0.001
*A. macrocephala* > *N. pernula*	5.18	<0.001
*H. arctica* > *Astarte* sp.	3.83	0.003
*Macoma* sp. > *Astarte* sp.	3.53	0.008

**Figure 4:**
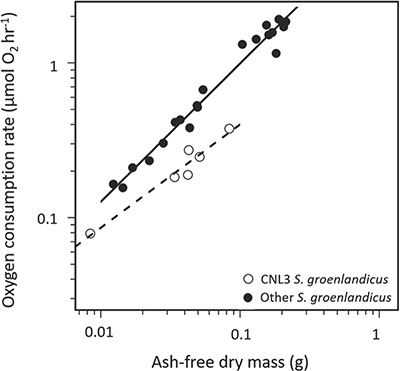
*Serripes groenlandicus* from sampling station CNL3 in open circles and individuals from all other stations in closed circles for both 2017 and 2018.

While *M*O_2_ of *S. groenlandicus* did not differ between sampling years, evidence of spatial variation was observed. Lower *M*O_2_ rates were recorded in individuals collected at station CNL3 compared to individuals from the other stations (Fig. 4). The slopes (F_1,22_ = 6.28, *P* = 0.020) and intercepts were significantly different (F_1,23_ = 79.98, *P* < 0.001).

**Figure 5:**
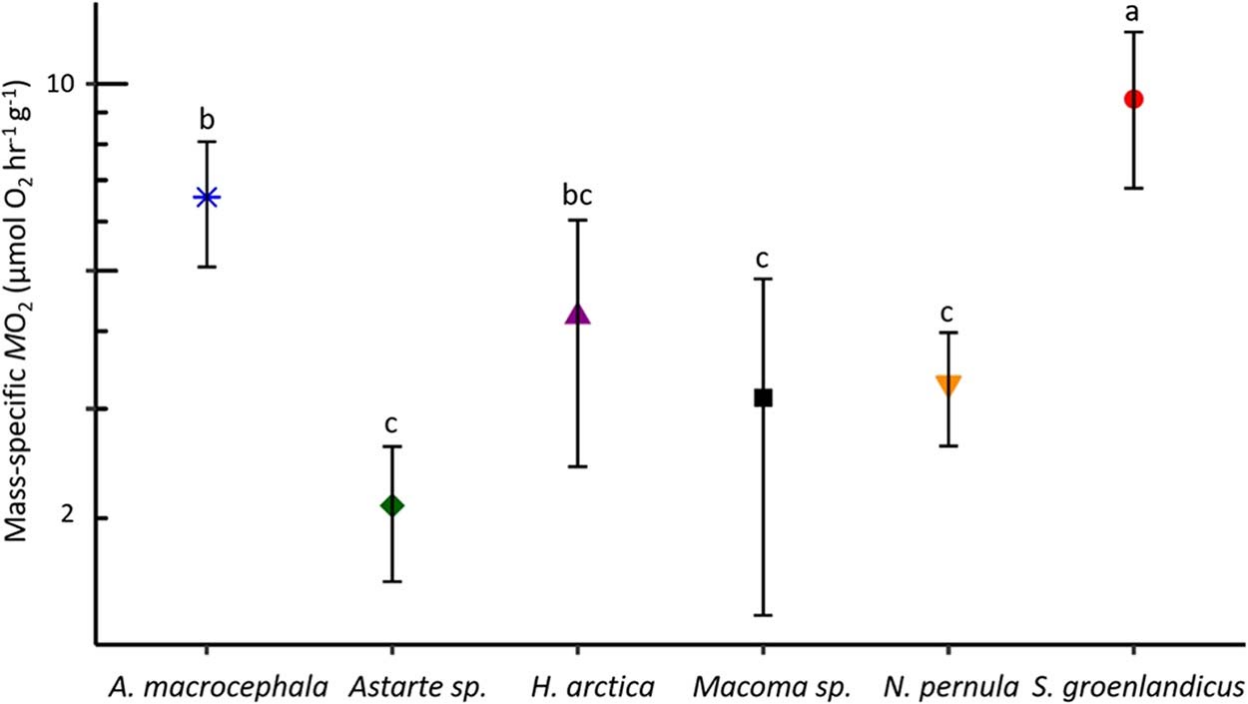
Average mass-specific respiration rates (μmol O_2_ hr^−1^ g^−1^) for each species with standard deviations represented by error bars. *Serripes groenlandicus* had a significantly higher rate than *Macoma* sp. (*t* = 9.6, *P* < 0.001), *Astarte* sp. (*t* = 6.8, *P* < 0.001), *N. pernula* (*t* = 7.3, *P* < 0.001), *H. arctica* (*t* = 4.3, *P* < 0.001) and *A. macrocephala* (*t* = 3.1, *P* = 0.028). The rate for *A. macrocephala* was significantly higher than *Macoma* sp. (*t* = 4.7, *P* < 0 0.001), *Astarte* sp. (*t* = 4.2, *P* < 0.001) and *N. pernula* (*t* = 3.8, *P* = 0.003).

Mass-specific respiration rates declined rapidly with increasing body size for all species except *S. groenlandicus* (Fig. 3b). The slope for *S. groenlandicus* was not significantly different from zero (*t* = −0.82, *P* = 0.42), while for all other taxa slopes ranged from −0.19 to −0.48. There were statistical differences in average mass-specific respiration rates among the species (F_5, 87_ = 25.75, *P* < 0.001; Fig. 5). *Serripes groenlandicus* had a significantly higher rate than the other five species, and the rate for *A. macrocephala* was significantly higher than *Macoma* sp., *Astarte* sp. and *N. pernula* (Fig. 5).

## Discussion

We measured oxygen consumption rates of six dominant macrofauna from the northern Bering and southern Chukchi Sea shelves to determine metabolic demand and organic carbon consumption. Overall, we observed taxonomic variability in metabolic demand with average mass-specific *M*O_2_ rates ranging from 2.1 to 9.5 μmol O_2_ hr^−1^ g^−1^, highlighting the need for species-specific measurements to improve estimates of organic carbon consumption by the benthos. Metabolic scaling coefficients (i.e. slope) also varied among species.

### Inter- and intraspecific variation in metabolic rates

We found species-specific standard metabolic rates (*M*O_2_), indicating a wide range in the amount of organic material that benthic species need to consume to maintain baseline metabolic function. For example, the metabolic demand of a hypothetical *S. groenlandicus* with 0.1 g AFDM would be 4.3 times higher than that of a similarly sized individual *Astarte* sp. These rates likely represent conservative estimates of organic carbon consumption. In polar regions, benthic organisms exhibit low metabolic rates when food availability is low, which then increase in response to phytodetrital inputs or elevated food concentrations (Brockington and Clarke, 2001; Sejr *et al.*, 2004).

For the bivalves, these differences in respiration rate among species may be related to the age or life stage of the individuals sampled. The individuals of all species used in our experiments were relatively similar in size (Table 2); however, the maximum achievable length observed in the field varies among species, such that incubated individuals may have been juveniles in some cases. We calculated the ratio of measured body length to maximum achievable length as a proxy to illustrate this relationship (Table 2). In particular, *S. groenlandicus* had the highest average *MO*_2_, but the lowest average measured length to maximum achievable length ratio of only 0.15. Individuals were mostly small, compared to their large maximum achievable size of up to 100 mm in shell length (Lubinsky, 1980; Kilada et al., 2007). In contrast, *Astarte sp*. had the lowest *MO*_2_ and the highest average length to maximum achievable length ratio of 0.64. *Astarte sp*. reach maximum lengths of only 30 mm, but rarely exceed 15 mm (Madsen, 1949; Schaefer et al., 1985), so our individuals were closer to their maximum size compared to *S. groenlandicus.* Overall, individuals selected were likely of different life stages and ages, which can impact respiration rates (Sukhotin and Pörtner, 2001). For instance, all *S. groenlandicus* individuals were smaller than 22 mm in length, which is smaller than the typical size at sexual maturity (Kilada et al., 2007), indicating these individuals were likely all juveniles. A more rapid growth rate that would be expected in these juveniles would thus contribute to the higher *MO*_2_ measured for this species.

In Young Sound, NE Greenland, the respiration rates of 26 individuals of *H. arctica* were measured at −1.3 °C with a constant food supply (Sejr *et al.*, 2004). Adjusting for the differences in temperature using Q_10_ = 3.64 (Peck and Conway, 2000) and feeding conditions (using an equation from Sejr *et al.*, 2004), *M*O_2_ for a 0.5 g *H. arctica* was 2 times higher in our study compared to that observed in Sejr *et al.* (2004). Conspecific metabolic rates vary due to numerous factors, such as genotype or environmental conditions during early life stages (Burton *et al.*, 2011). The discrepancies between the rates measured in these studies could also be due to temperature compensation (Rastrick and Whiteley, 2011) or other factors related to differences in experimental design.

The metabolic scaling coefficient, which relates metabolic rate to body mass, is broadly estimated to be 0.75 in a wide variety of taxa (Kleiber, 1932). However, deviations from the ‘3/4-power law’ occur for a variety of reasons in both intra- and interspecific metabolic studies (Glazier, 2005). The metabolic scaling coefficient was close to 0.75 for three of the species measured here, *H. arctica*, *Astarte* sp. and *A. macrocephala* (Table 2), but was much higher for *S. groenlandicus* (b = 0.94) and lower for *Macoma* sp. (b = 0.52). Here again, life stage may be a factor for the high metabolic scaling coefficient of *S. groenlandicus*. Metabolic scaling is often higher in juveniles compared to adults, likely due to greater energetic demands of rapid growth as opposed to somatic tissue maintenance (Glazier, 2005). In 2017, we additionally measured the respiration rates of four large *S. groenlandicus* individuals ranging from 40.6 to 60.0 mm length, which were likely mature adults (Supplementary Figure 2). When *M*O_2_ was calculated for pooled juvenile and adult individuals, the scaling coefficient declined from b = 0.94 to b = 0.85, suggesting the rate of change of respiration rate with increasing body mass is higher for juveniles than adults (adults-only exponent was b = 0.81; Supplementary Figure 2). Additionally, the slope of the mass-specific oxygen consumption rate for *S. groenlandicus* juveniles was not significantly different from zero, suggesting that the mass-specific respiration rate does not change with increasing biomass. Ontogenetic shifts are known to occur in mass-specific metabolic scaling from near isometry (b = 0) to allometry (b < 0), relating to changes in body shape (Glazier *et al.*, 2015).

### Potential environmental effects on metabolic rate

Although we did not sample with the intent to evaluate interannual variability in metabolic rates, we were able to compare data from two years for two species. The small temperature variation in our treatments for each year of about 0.3 °C complicates interpretation of this result given the direct effect of temperature on metabolic rate (Peck *et al.*, 2002; Clarke and Fraser, 2004; Trigos *et al.*, 2015). Nonetheless, the respiration rates of *S. groenlandicus* were not significantly different between years, suggesting the temperature difference did not affect our results. Relative thermal independence of metabolic rate has been observed in other benthic species. For instance, the respiration rate of the amphipod *Anonyx nugax* remained constant over the temperature range 1–3 °C, suggesting metabolic adaptation to natural variability in environmental conditions (Opalinski and Weslawksi, 1989). In contrast, *M*O_2_ of *Macoma* sp. was significantly higher in 2018. However, if the increased respiration rates were strictly due to temperature, the effect we observed would indicate a Q_10_ of 36 730, which is well beyond typical values (McMahon and Wilson, 1981; Peck and Conway, 2000), suggesting other factors produced this result. Most individuals were collected at different stations in each year, making it difficult to tease apart spatial from temporal differences in the environment as possible influences. Total organic carbon (TOC) concentration was roughly five times higher at station DBO3.8 where most individuals were collected in 2018, compared to station CNL3 where most individuals were collected in 2017, but TOC values were not substantially different between years at either station (Mincks unpublished data). In contrast, chlorophyll-a concentrations in surface sediments were substantially higher in 2017 than in 2018 at both stations due to the timing of ice retreat. Thus, the feeding environment *in situ* may have played a role in producing the interannual differences in *M*O_2_ for *Macoma* sp. Alternatively, this species may simply lack temperature compensation (cf., Rastrick and Whiteley, 2011). Regardless, the experimental temperature difference between the two years is small compared to the seasonal and interannual fluctuations experienced in the region (Danielson *et al.*, 2020).

While the *MO*_2_ of *S. groenlandicus* did not differ between years, evidence of spatial variation was observed, with individuals from one sampling station (CNL3; Fig. 1) exhibiting lower *MO*_2_ compared to individuals from the other stations. This difference may reflect physiological differences related to environmental factors. Intraspecific variation in respiration rate can be related to a variety of factors, such as environmental conditions during early development (Burton et al., 2011). Growth rate of *S. groenlandicus* also varies spatially due to environmental conditions, which likely reflect variations in trophic conditions, and has thus been proposed as an indicator of environmental change (Ambrose et al., 2006; Kilada et al., 2007; Carroll et al., 2009; Gerasimova et al., 2019). While the average depth and other physical variables did not vary substantially at the sampling locations where *S. groenlandicus* was collected (Table 1), sandier sediment and a lower C: N ratio were observed at station CNL3 compared to the other locations (Mincks unpublished data). Both of these variables may reflect feeding conditions, potentially as a function of hydrodynamics at this site where current speeds are high due to the constriction of flow through the Bering Strait (Danielson et al., 2014). The reduced metabolic rate at the sandier CNL3 site seems to contradict evidence of a slower growth rate at stations with high silt fraction reported elsewhere (Gerasimova et al., 2019). However, growth rates and basal metabolic rates do not always align (Sebens, 2002). This spatial difference highlights a need to measure respiration rates from across the region of interest. Individuals with low respiration rates may be buffered against environmental conditions due to their low maintenance costs, which may yield greater fitness in poor trophic conditions (Burton et al., 2011). Not accounting for spatial variability in metabolic rate may bias modelling estimates of regional carbon demand and food web dynamics.

In contrast to *S. groenlandicus*, *Macoma* sp. collected from station CNL3 showed no clear impact of station on respiration rate. However, growth rate of *Macoma* sp*.* may be less sensitive to environmental conditions than *S. groenlandicus* (Gerasimova *et al.*, 2019) and may be buffered against environmental variability.

### Implications for benthic ecosystem functioning

Environmental changes are already resulting in temperature increases, changes in primary production and shifts in benthic species composition, structure and biomass. Species-specific respiration rates suggest these changes will alter organic matter processing and carbon flow pathways in the Pacific Arctic benthos.

Metabolic rate increases with increasing temperature up to an optimal range. Respiration rates were used to estimate the expected increase in metabolic demand of each taxonomic group at a projected future temperature of 5 °C (Mora *et al.*, 2013) assuming Q_10_ values between 2.56 and 3.64 (Peck and Conway, 2000) following the equation:

$$ {\mathrm{R}}_2={\mathrm{R}}_1{{\mathrm{Q}}_{10}}^{\frac{{\mathrm{T}}_2-{\mathrm{T}}_1}{10}} $$

where R_1_ is the measured respiration rate at the initial temperature (T_1_ = 0.9 °C) and R_2_ is the calculated respiration rate at the projected temperature (T_2_ = 5 °C). These calculated rates provide an estimate of the increase in carbon demand under projected future warming scenarios. Indeed, bottom-water temperatures of 4 °C are already occurring in the Bering Strait region (Huntington *et al.*, 2020). At a projected future temperature of 5 °C, average mass-specific *M*O_2_ would increase by 48–70% to a value of 14.0–16.1 μmol O_2_ hr^−1^ g^−1^ for *S. groenlandicus*, 9.7–11.2 for *A. macrocephala*, 6.2–7.2 for *H. arctica*, 4.8–5.6 for *N. pernula*, 4.6.-5.3 for *Macoma* sp. and 3.1–3.6 for *Astarte* sp., again assuming Q_10_ values between 2.56 to 3.64 (Peck and Conway, 2000) and that an upper critical temperature limit has not been exceeded (Peck *et al.*, 2002). However, Q_10_ likely varies among the species and over different temperature ranges.

With this increase in standard metabolic demand and a potential decline in phytodetrital input to the seafloor (Lee *et al.*, 2013; Moore and Stabeno, 2015; Lovvorn *et al.*, 2016), carbon reserves in the sediment may become depleted, although there are some projections of increased primary production and input to the seafloor in this region (Grebmeier *et al.*, 2015a). Temperature-induced increases in metabolic demand coupled with low food availability can result in reproductive failure, death and a subsequent decline in benthic production and biomass (Hummel *et al.*, 2000). If input of organic carbon to the benthos declines and carbon resources in the sediments are depleted, biomass of bivalves and amphipods in persistent macrobenthic hotspots may then decline, with deleterious impacts on upper trophic levels, such as benthic-feeding marine mammals and birds that depend on these prey items. For instance, in the northern Bering Sea shelf, decline of the spectacled eider population has been associated with a reduction in the biomass of bivalve populations that serve as critical prey for these birds (Lovvorn *et al.*, 2009).

However, species with low metabolic demand may be more adapted to this low-food future scenario. Nuculanidae (which includes *N. pernula*) currently dominate in the northern region of our study area (Grebmeier *et al.*, 2015a). The relatively low respiration rate, and thus low metabolic demand, of *N. pernula* may leave it preadapted to the lower-productivity waters of this area, which is influenced by the Alaska Coastal Current. This low metabolic demand may confer a physiological competitive advantage over other taxonomic groups with higher carbon requirements (Burton *et al.*, 2011; McClain *et al.*, 2020). Therefore, we hypothesize that species with low metabolic rates, such as *Astarte* sp. and *N. pernula*, may dominate under a low-food scenario given their reduced organic carbon requirements necessary to maintain metabolic function. In contrast, species with high metabolic rates, such as *S. groenlandicus* and *A. macrocephala*, may be hindered by higher carbon demands and become food limited. In response to ocean warming, spatial shifts in the frequency and abundance of species associated with differing physiological tolerances has already been identified in many other regions (Sunday *et al.*, 2012).

In the Arctic, emerging evidence indicates environmental change has influenced the distribution of macrofaunal biomass, with declining biomass in some areas and increasing biomass in others (Moore *et al.*, 2018; Goethel *et al.*, 2019). In addition to changes in overall biomass, shifts in community structure and composition are occurring (Grebmeier, 2012; Waga *et al.*, 2020). Shifts in dominant species could impact community metabolic demand even if total biomass remained constant. For instance, if *S. groenlandicus* were outcompeted and replaced by *N. pernula*, carbon demand would decline given the lower *M*O_2_ of *N. pernula*.

In conclusion, the average mass-specific *M*O_2_ of sampled species ranged from 2.1 to 9.5 μmol O_2_ hr^−1^ g^−1^, with species-specific differences up to 4.3 times for a 0.1 g AFDM individual. These differences in *M*O_2_ have implications for the overall carbon demand of the benthic infaunal community as assemblages are likely to continue to change under future climate scenarios.

## Supplementary material

Supplementary material is available at *Conservation Physiology* online.

## Funding

This research was part of the Arctic Integrated Ecosystem Research Program (IERP; http://www.nprb.org/arctic-program/). This manuscript is Publication ArcticIERP-17. Funding for the program was provided by the North Pacific Research Board, US Bureau of Ocean and Energy Management, Collaborative Alaskan Arctic Studies Program and US Office of Naval Research. Generous in-kind support for the program was contributed by the US National Oceanic Atmospheric Administration Alaska Fisheries Science Center and Pacific Marine Environmental Laboratory, University of Alaska Fairbanks, US Fish and Wildlife Service and US National Science Foundation. Support for this research was also provided by the Robert and Kathleen Byrd Award. This publication was, in part, made possible by the University of Alaska Fairbanks Office of the Vice Chancellor for Research Publication Award.

## Supplementary Material

Supplementary_material_coab007
