# Supplementary material for: Changes to benthic community structure may impact organic matter consumption on Pacific Arctic shelves
Source: Conserv Physiol. 2021 Mar 31;9(1):coab007. doi: 10.1093/conphys/coab007 (PMC8015422; doi:10.1093/conphys/coab007)
Supplement: Supplementary_material_coab007 [file supplementary_material_coab007.docx]

Supplementary material


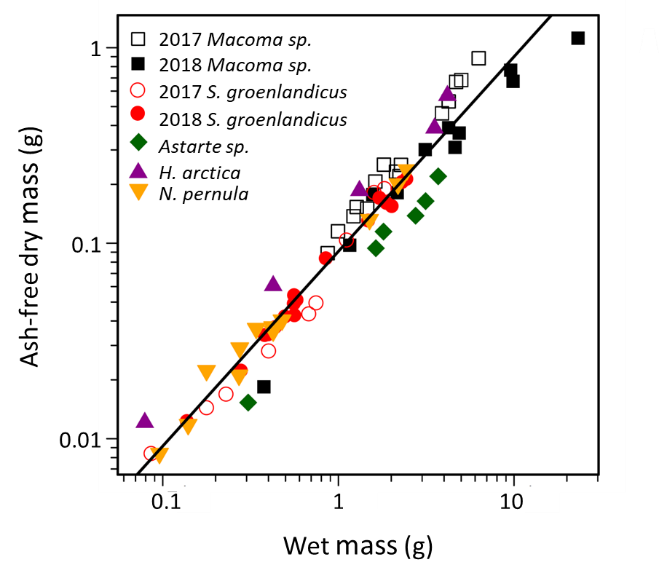

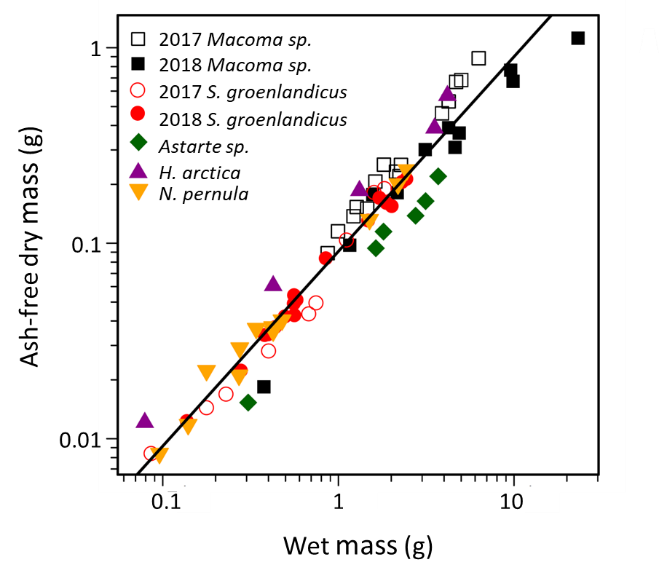

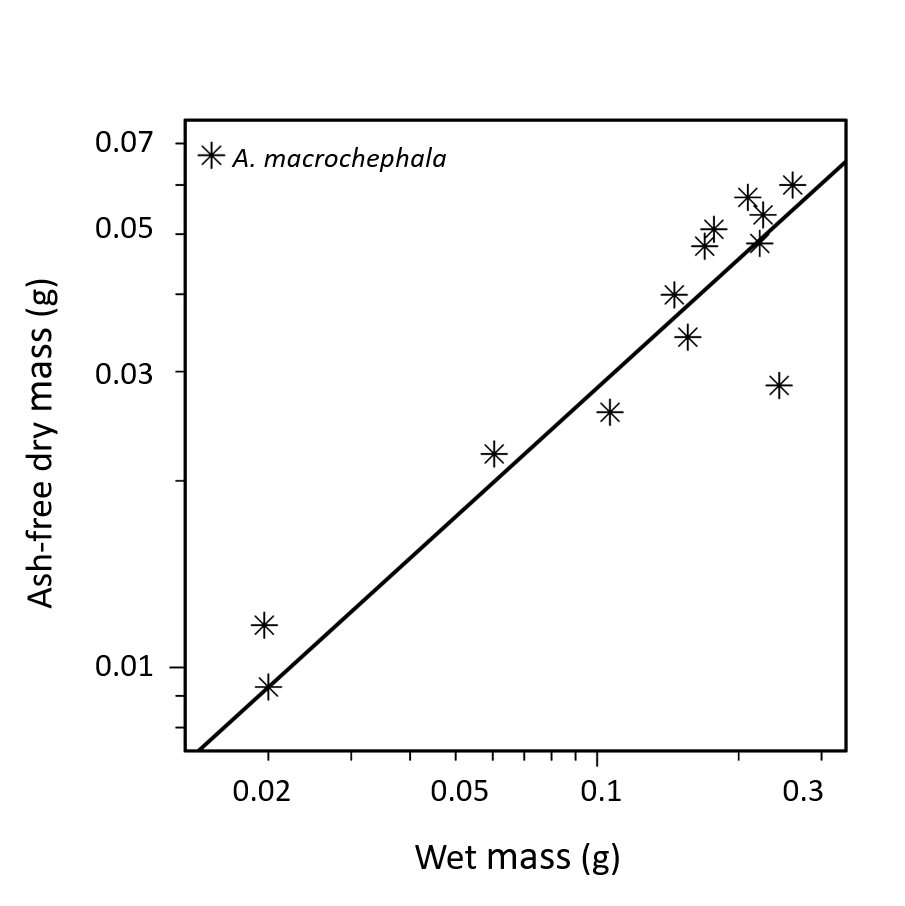


Supplementary Figure 1: (a) Relationship between wet mass of whole bivalve including the shell (g) and the ash-free dry mass of the tissue without the shell (g) of all bivalves incubated. Power function is y = 0.09x^1.00^ (n = 75, R^2^ = 0.95, p < 0.001). 2017 *Macoma* sp. (black open squares, n = 15), 2018 *Macoma* sp. (black squares, n = 11), 2017 *Serripes groenlandicus* (red open circles, n = 9), 2018 *Serripes groenlandicus* (red circles, n = 18), *Astarte* sp. (green diamonds, n = 6), *Hiatella arctica* (purple upright triangles, n = 5), and *Nuculana pernula* (yellow upside-down triangles; n=12). (b) Relationship between wet mass (g) and the ash-free dry mass (g) of whole *Ampelisca macrocephala*. Power function is y = 0.14x^0.69^ (n = 14, R^2^ = 0.90, p < 0.001).


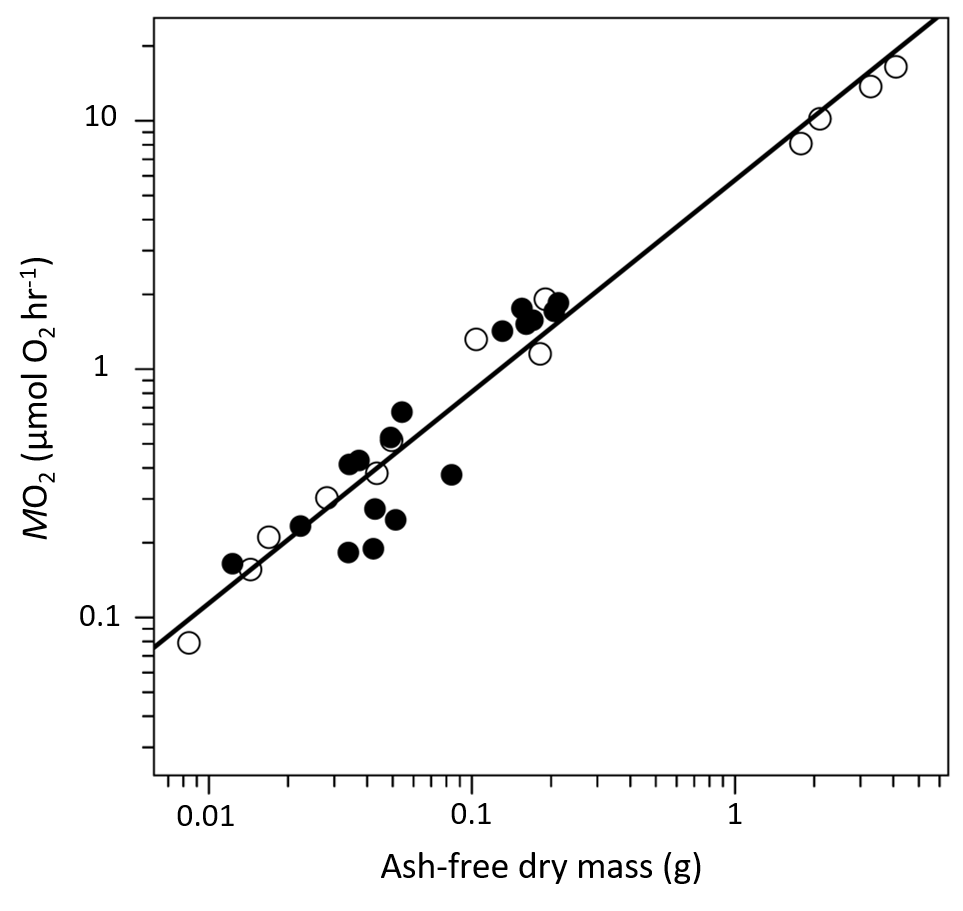


Supplementary Figure 2: Oxygen uptake rate (µmol O_2_ h^-1^) versus ash-free dry mass (g) for *Serripes groenlandicus* (2017 individuals in open circles and 2018 individuals in closed circles) with 26 juveniles and 4 adults. Power function is R = 5.77mass^0.85^ (R^2^ = 0.95, p < 0.001).
